# Supplementary material for: Moyamoya Vasculopathy in Neurofibromatosis Type 1 Pediatric Patients: The Role of Rare Variants of RNF213
Source: Cancers (Basel). 2023 Mar 22;15(6):1916. doi: 10.3390/cancers15061916 (PMC10047491; doi:10.3390/cancers15061916)
Supplement: Supplementary file 1 [file cancers-15-01916-s001.zip › cancers-2256473-supplementary.pdf]

Supplementary data S1

NF1 cDNA PCR primers

FRAGMENT	FW	REV
F1	ACAGACCCTCTCCTTGCCTC	GCAACTGTTTGACCAGCATCG
F2	TGAGCACAACAAGGAATGTCTAATC	TGGTACAGTTTTGTAAATTCATCTGG
F3	ACGACTCCTGAAGGAAACAGC	CTGACTGCCTCTTGAGAATGG
F4	TGGCCATGGAGGAAGTAGG	GGTGTGCTCCACAACCTTG
F5	ATCTGCCTGGCTCAGAATTC	CAGAGCCTCCATTGCTTCC
F6	ATTACAGGGCTCGTCCAACTG	AGTTTGGTCTGGGCTTGTCG
F7	ACGGGGTAGGATGTGATATTCC	TCCCAAGCCTCAGTGTTTCC
F8	GGAAGCAGATATCCGGTGTGG	GGATACAGAGCAGGACTCAA
F9	CATGGGTCCAGTCAGTGAAC	GCCTGGTCCAAATCTCTTGT
F10	GGGCAAGCTAGCATTGAAAC	CCACCTGTTTGCGCACTTTC
F11	GTCTTACAAGAGATTTGGACCA	GGAACCACATTGGCCAGAGC
F12	GCACAGAATTTGACACACTTGC	AGGTTCCGCTGGTTTTCC
F13A	TCTCTTCCGAGGCAACAGC	TTTATTCAGTAGGGAGTGGCA
F13B	AACTTGCCACTCCCTACTG	GCAAGCACATTGCCGTCACT
F14	AATCCTGCCATTGTCTCACC	GGCTGGACCAGTGTGTATCT
F15	CCAGCAACAGGGATCATAAAGC	CTTTGAGGCCAGTCAGCAGC
F16	CTGGCTTTGCTTACGACAACG	GGCCGAATCTTGGTGTGTTGG
F17	TTCACCTTAACCATTGCAAACC	GGTGTTTCAATTCAATACTAGATTTGC
F18	CCAATGAGCCACACCTCACG	GGACAGCATCAGCATGTAGCG
F19	ATTGGTTTCAAGCAAGGTTATTG	TGCATGCCTCCATGATCTCC
F20	ACAGGTCATTCTCTCCTGGC	TCCGGATTGCCATAAATACTTCC
F21	TGTGCTGCAGCTTGATGAGG	TTGGATAGGTGGCTGCAAGG
F22	CATTCATCATGGTGACCCTTCC	GAAACACAACACTGGCCTCTGC
F23	GCGCTGCTTCTTACTGTTCTAGC	GCTGGGAAGTTGCAAGTGAGG
F24	TCTTGATGCCTTGATTGACACG	AAGGACAGGGAAGGGGTCAC
